# Self-Organizing Map-Based Assessment of Immune-Related Adverse Events Caused by Immune Checkpoint Inhibitors

**DOI:** 10.7759/cureus.76813

**Published:** 2025-01-02

**Authors:** Satoshi Nakao, Koumi Miyasaka, Mika Maezawa, Kohei Shiota, Mari Iwata, Sakiko Hirofuji, Nanaka Ichihara, Moe Yamashita, Yuka Nokura, Kana Sugishita, Tomofumi Yamazaki, Hirofumi Tamaki, Takeshi Hirota, Mayako Uchida, Kazuhiro Iguchi, Mitsuhiro Nakamura

**Affiliations:** 1 Laboratory of Drug Informatics, Gifu Pharmaceutical University, Gifu, JPN; 2 Department of Pharmacy, Kyushu University Hospital, Fukuoka, JPN; 3 Department of Pharmacy, Yanaizu Pharmacy, Gifu, JPN; 4 Laboratory of Community Pharmacy, Gifu Pharmaceutical University, Gifu, JPN

**Keywords:** immune checkpoint inhibitor (ici), immune-related adverse event (irae), japanese adverse drug event report database, self-organizing map, spontaneous reporting system

## Abstract

Introduction

Remarkable progress has been made in the field of cancer therapy in recent years owing to the development of immune checkpoint inhibitors (ICIs); however, controlling immune-related adverse events (irAEs) remains challenging for treatment completion. This is the first study to visualize the irAE profiles of ICIs using self-organizing maps (SOM) and to combine this with decision tree analysis. The purpose of this study is to identify adverse events from a wide variety of irAEs in eight ICIs that can be useful for early detection.

Methods

Three anti-programmed death-1, three anti-programmed death-ligand 1, and two anti-cytotoxic T-lymphocyte antigen-4 antibodies were analyzed. Reported irAEs extracted from the Japanese Adverse Drug Event Report (JADER) database were analyzed based on the preferred term in the Medical Dictionary for Regulatory Activities. SOM was applied using the SOM package in R (version 4.1.2; R Foundation for Statistical Computing, Vienna, Austria).

Results

The JADER database registered 880,999 reports published between April 2004 and February 2024. The numbers of irAEs reported for atezolizumab, avelumab, cemiplimab, durvalumab, ipilimumab, nivolumab, pembrolizumab, and tremelimumab were 3797, 361, 17, 2554, 9315, 16,574, 11,487, and 196, respectively. After ICIs were classified using the SOM, they were adapted for decision tree analysis. The eight ICIs were divided into four groups based on the reported rates of type 1 diabetes mellitus and hematological disorders.

Conclusion

Our findings provide a reference for healthcare providers to predict irAE characteristics induced by ICIs in patients, thereby facilitating effective cancer treatment.

## Introduction

In recent years, remarkable progress has been made in cancer treatment owing to the widespread use of immunotherapy [1,2]. Immune checkpoint molecules, including cytotoxic T-lymphocyte antigen-4 (CTLA-4) and programmed death-1 (PD-1), are involved in the regulation of peripheral tolerance to prevent autoimmunity [3]. Cancer cells interfere with immune checkpoint control mechanisms and evade detection by the immune system for continued proliferation and metastasis [1]. Immune checkpoint inhibitors (ICIs) are monoclonal antibodies that act on CTLA-4, PD-1, and its ligand PD-L1 to remove cancer cells and restore the immune system [3-5].

Immune-related adverse events (irAEs) caused by ICIs differ from those caused by cytotoxic or molecular targeted agents. The time to onset of toxicity is slow and does not follow the cyclic pattern of conventional cytotoxic agents. The mechanism underlying toxicity is not yet understood and may vary across patients, even for the same drug [6].

Adverse effects associated with ICIs can affect any organ and may result from the activation of autoreactive T-cells, which damage host tissues [4]. These irAEs most commonly affect the colon, liver, lungs, pituitary gland, thyroid, and skin but rarely affect the heart, nervous system, and other organs [4,7,8]. Individual irAE profiles vary by organ, exhibiting autoimmune-like activity [9]. This depends on the class of ICI drugs used (PD-1/PD-L1 vs. CTLA-4). Compared with PD-1/PD-L1 inhibitors, CTLA-4 inhibitors are more likely to cause colitis, renal hypofunction, and dermatitis and are less likely to cause pneumonia, hypothyroidism, and skeletal symptoms such as myalgia and arthralgia [6,9,10].

Classifying the complex adverse events (AEs) among irAEs caused by ICI is important to enable early irAE detection by healthcare providers. However, there is a lack of information classifying ICIs based on the pattern of AEs. Studies using the Spontaneous Reporting System (SRS) may visualize the results of many drugs and AEs in a format that is easy for clinicians to understand and use. Examples of visualization were as follows: a dendrogram classifying various anticancer drugs based on various AEs of drug-induced hand-foot syndrome by cluster analysis [11]; a report classifying various psychotic drugs based on various AEs of malignant syndromes and visualizing them in a dendrogram and visualizing the clinical characteristics of each cluster obtained by using a cluster mean plot [12]; reports visualizing the reported odds ratio, which is a pharmacovigilance index of AEs of SRS, in a heat map [13,14]; visualization of association rules for the antecedents and the consequences of AEs using the Package arulesViz for association analysis [15,16]; and visualization of the association between reporting odds ratio and p-value using a volcano plot [17]. While these visualization techniques are powerful for identifying overall trends at a glance, they may be difficult for clinicians to use for early-intervention decision-making when faced with individual AEs.

Kohonen et al. reported a promising pattern recognition method, namely, a self-organizing map (SOM) [18], which can create an easy-to-understand, visual, two-dimensional map reflecting the data structure of adverse drug reaction (ADR) information across drugs [19]. SOM has been used to visualize the side effects of anticancer agents, neuroleptic malignant syndrome, and rhabdomyolysis [20-22]. Visualization of AEs using SOM is expected to improve medical safety by tracking the trends in the occurrence and avoidance of ADRs, prediction of new adverse drug reactions, and consideration of alternative drugs in the real-world clinical setting [19].

The SRS for AEs is crucial for the safety evaluation of drugs based on AE signals obtained by mining the SRS database [23]. Competitive learning using SOM classifies the data into the appropriate number of units and then uses decision tree analysis to automatically generate rules to describe the relationships among the units. This is the first study to visualize the irAE profiles of ICIs using SOM and to combine this with decision tree analysis. The purpose of this study is to obtain rules that explain the relationship between ICI and irAE through SOM and decision tree analysis. The rules obtained can facilitate the detection and intervention of irAEs at an early stage by healthcare professionals.

## Materials and methods

Data source

The data source for AEs was the Japanese Adverse Drug Event Report (JADER) database, which was collected and fully anonymized by the Pharmaceuticals and Medical Devices Agency (PMDA). The AE reports recorded in this database were downloaded from the PMDA website [24]. This database consists of the following four tables: (1) DEMO (patient information such as sex, age, and weight), (2) DRUG (drug information such as generic name, starting date of administration, and dosage, and drug involvement, which classifies drug involvement in AEs as “suspected drug,” “concomitant drug,” and “interaction”), (3) REAC (information on AE classification and date of occurrence), and (4) HIST (information on the underlying disease). This database structure was designed based on the International Council on Harmonization E2B guidelines. In this study, we extracted and analyzed the “suspected drug” records. For data table integration, a relational database was constructed using FileMaker Pro version 20.3.2 (Claris International Inc., Santa Clara, CA, USA).

Definition of adverse events

AEs were defined using the preferred term (PT) in the Medical Dictionary for Regulatory Activities, Japanese version 23.1 [25]. We used 125 PTs related to irAEs (Table 1).

**Table 1:** Number of adverse events for each preferred term associated with immune-related adverse events by immune checkpoint inhibitors “Case” represents the number of irAE reported for eight immune checkpoint inhibitors.“Total” represents the number of irAE reported for all drugs, including eight immune checkpoint inhibitors.

irAE Categories	Preferred terms	Preferred term code	Case (n)	Total (n)	Reporting Ratio (%)
Adrenal insufficiency					
	Acute adrenocortical insufficiency	−	142	241	58.9
	Addison's disease	10001130	32	42	76.2
	Adrenal insufficiency	−	2858	3621	78.9
	Adrenocorticotropic hormone deficiency	−	629	662	95.0
	Glucocorticoid deficiency	−	2	15	13.3
	Immune-mediated adrenal insufficiency	−	472	472	100.0
	Primary adrenal insufficiency	−	55	70	78.6
	Secondary adrenocortical insufficiency	−	523	611	85.6
	Steroid withdrawal syndrome	10042028	3	102	2.9
Colitis					
	Autoimmune colitis	10075761	62	63	98.4
	Colitis	10009887	1078	1519	71.0
	Colitis erosive	10058358	1	9	11.1
	Colitis ischaemic	10009895	22	1117	2.0
	Colitis microscopic	10056979	30	719	4.2
	Colitis ulcerative	10009900	120	992	12.1
	Diarrhea	10012735	1250	11910	10.5
	Diarrhoea haemorrhagic	10012741	4	61	6.6
	Enteritis	10014866	74	267	27.7
	Enterocolitis	10014893	562	2147	26.2
	Enterocolitis hemorrhagic	10014896	18	971	1.9
	Inflammatory bowel disease	10021972	4	54	7.4
	Neutropenic colitis	10062959	1	71	1.4
Encephalitis/Meningitis					
	Encephalitis	10014581	285	754	37.8
	Encephalitis autoimmune	10072378	134	237	56.5
	Immune-mediated encephalitis	10083074	107	112	95.5
	Meningitis	10027199	151	648	23.3
Eye disease					
	Autoimmune uveitis	10075690	1	1	100.0
	Immune-mediated uveitis	10083069	66	66	100.0
	Uveitis	10046851	250	846	29.6
Gastritis					
	Gastritis	10017853	39	221	17.6
	Immune-mediated gastritis	10084296	54	54	100.0
Hematological disorder					
	Agranulocytosis	10001507	65	4359	1.5
	Aplasia pure red cell	10002965	33	608	5.4
	Autoimmune hemolytic anemia	10073785	80	380	21.1
	Febrile neutropenia	10016288	943	11781	8.0
	Immune thrombocytopenia	10083842	233	1353	17.2
	Neutropenia	10029354	469	13328	3.5
	Neutrophil count decreased	10029366	726	17276	4.2
	Platelet count decreased	10035528	616	21368	2.9
	Thrombocytopenia	10043554	255	7545	3.4
	Thrombocytopenic purpura	10043561	8	545	1.5
Hemophagocytic syndrome					
	Haemophagocytic lymphohistiocytosis	10071583	245	1389	17.6
Hepatitis					
	Acute hepatic failure	10000804	29	629	4.6
	Alanine aminotransferase increased	10001551	260	3996	6.5
	Aspartate aminotransferase increased	10003481	272	3715	7.3
	Autoimmune hepatitis	10003827	104	549	18.9
	Drug-induced liver injury	10072268	471	5968	7.9
	Hepatic enzyme increased	10060795	96	1039	9.2
	Hepatic failure	10019663	169	1655	10.2
	Hepatic function abnormal	10019670	1633	20649	7.9
	Hepatitis	10019717	245	763	32.1
	Hepatitis acute	10019727	17	1113	1.5
	Hepatotoxicity	10019851	43	235	18.3
	Immune-mediated hepatic disorder	10083521	775	783	99.0
	Immune-mediated hepatitis	10078962	272	275	98.9
	Liver disorder	10024670	862	12066	7.1
	Liver function test abnormal	10024690	4	627	0.6
	Liver injury	10067125	25	240	10.4
	Transaminases increased	10054889	10	193	5.2
Hyperthyroidism					
	Hyperthyroidism	10020850	1141	2069	55.1
	Immune-mediated hyperthyroidism	10083517	197	197	100.0
	Primary hyperthyroidism	10075899	1	2	50.0
	Thyrotoxic crisis	10043786	25	114	21.9
	Toxic nodular goitre	10044242	1	3	33.3
Hypopituitarism					
	Hypophysitis	−	350	362	96.7
	Hypopituitarism	−	616	673	91.5
	Immune-mediated hypophysitis	−	92	93	98.9
Hypothyroidism					
	Autoimmune hypothyroidism	10076644	33	33	100.0
	Central hypothyroidism	−	47	345	13.6
	Hypothyroidism	10021114	2981	4587	65.0
	Immune-mediated hypothyroidism	10083075	831	832	99.9
	Primary hypothyroidism	10036697	19	28	67.9
Myasthenia gravis					
	Myasthenia gravis	10028417	515	774	66.5
Myocarditis					
	Autoimmune myocarditis	10064539	6	6	100.0
	Eosinophilic myocarditis	10014961	1	57	1.8
	Immune-mediated myocarditis	10082606	208	215	96.7
	Myocarditis	10028606	498	1846	27.0
Myositis/Rhabdomyolysis					
	Autoimmune myositis	10082418	14	19	73.7
	Immune-mediated myositis	10083073	199	372	53.5
	Myositis	10028653	401	593	67.6
	Polymyositis	10036102	60	179	33.5
	Rhabdomyolysis	10039020	209	7668	2.7
Nephritis/renal dysfunction					
	Acute kidney injury	10069339	407	9890	4.1
	Autoimmune nephritis	10077087	11	24	45.8
	Glomerulonephritis	10018364	10	113	8.8
	Glomerulonephritis acute	10018366	5	35	14.3
	Glomerulonephritis rapidly progressive	10018378	24	272	8.8
	Immune-mediated nephritis	10083070	45	46	97.8
	Immune-mediated renal disorder	10083522	112	112	100.0
	Nephritis	10029117	60	230	26.1
	Renal disorder	10038428	192	3113	6.2
	Renal failure	10038435	136	3085	4.4
	Renal impairment	10062237	984	15205	6.5
	Tubulointerstitial nephritis	10048302	481	2891	16.6
	Tubulointerstitial nephritis and uveitis syndrome	10069034	1	100	1.0
Neurological disorder					
	Guillain-Barre syndrome	10018767	114	884	12.9
Pancreatitis					
	Autoimmune pancreatitis	10069002	78	120	65.0
	Immune-mediated pancreatitis	10083072	116	116	100.0
	Pancreatitis	10033645	254	1645	15.4
	Pancreatitis acute	10033647	100	2561	3.9
Pneumonitis					
	Interstitial lung disease	10022611	7586	38932	19.5
	Pneumonia	10035664	1201	17751	6.8
	Pneumonitis	10035742	1976	3357	58.9
Rash					
	Erythema	10015150	130	6011	2.2
	Erythema multiforme	10015218	583	7006	8.3
	Oculomucocutaneous syndrome	10030081	9	1124	0.8
	Pemphigoid	10034277	364	2701	13.5
	Pruritus	10037087	156	3827	4.1
	Pruritus allergic	10063438	1	4	25.0
	Rash	10037844	935	12818	7.3
	Rash erythematous	10037855	32	437	7.3
	Rash macular	10037867	1	54	1.9
	Rash maculo-papular	10037868	74	318	23.3
	Rash papular	10037876	12	183	6.6
	Rash pruritic	10037884	33	240	13.8
	Toxic epidermal necrolysis	10044223	133	3672	3.6
Thyroid dysfunction					
	Autoimmune thyroid disorder	10079165	4	5	80.0
	Autoimmune thyroiditis	10049046	66	171	38.6
	Immune-mediated thyroiditis	10083071	94	94	100.0
	Thyroid disorder	10043709	115	193	59.6
	Thyroiditis	10043778	313	475	65.9
Type 1 diabetes mellitus					
	Diabetic ketoacidosis	10012671	256	1359	18.8
	Fulminant type 1 diabetes mellitus	10072628	617	791	78.0
	Latent autoimmune diabetes in adults	10066389	2	16	12.5
	Type 1 diabetes mellitus	10067584	880	1320	66.7

Target drugs

Between 2014 and early 2023, eight ICIs were available in Japan, including the anti-PD-1 antibodies nivolumab, pembrolizumab, and cemiplimab; anti-PD-L1 antibodies avelumab, atezolizumab, and durvalumab; and anti-CTLA-4 antibodies ipilimumab and tremelimumab (Table 2).

**Table 2:** Number of adverse events associated with immune-related adverse events of immune checkpoint inhibitors and unit number assigned to the 6 × 1 map created by the self-organizing map

Drug	Case (n)	Total (n)	Reporting Ratio (%)	Unit
Atezolizumab	3797	6692	56.7	4
Avelumab	361	597	60.5	5
Cemiplimab	17	31	54.8	5
Durvalumab	2554	3568	71.6	5
Ipilimumab	9315	12016	77.5	3
Nivolumab	16574	22944	72.2	2
Pembrolizumab	11487	17380	66.1	3
Tremelimumab	196	298	65.8	5

SOM

SOM is a type of unsupervised learning - a neural network model that visualizes and clusters the structure of data by self-organizing high-dimensional input data onto a low-dimensional map. SOM is based on competitive learning, where each neuron competes with input data on how similar it is to the input data, and the most similar neuron learns that data. In addition, the weights of the neurons around that neuron are also updated, forming a structure in which neurons with high similarity are placed close to each other, whereas neurons with low similarity are placed farther away [18].

When using data mining methods, such as SOM, it is generally important to consider all AEs without omissions. In this study, we categorized the 125 irAE PTs by irAE category (Table 1), with the percentage of reported irAEs in each category and a list of associated drugs on a two-dimensional map (Table 3). We then performed competitive learning by placing 125 AEs in layer 1 (input layer) and 100 units in layer 2 (output layer) in a 5 × 5 format, which was sufficiently greater than the number of drugs (eight categories).

**Table 3:** Reporting ratios (%) of the immune-related adverse events category associated with immune checkpoint inhibitors

irAE Categories	Atezolizumab	Avelumab	Cemiplimab	Durvalumab	Ipilimumab	Nivolumab	Pembrolizumab	Tremelimumab
Adrenal insufficiency	5.2	0.9	0.0	0.8	36.4	51.6	22.2	0.2
Colitis	1.5	0.2	0.0	0.5	6.1	9.5	4.4	0.3
Encephalitis/Meningitis	11.3	0.1	0.0	1.3	9.3	14.8	11.5	0.1
Eye disease	0.8	0.0	0.0	0.2	14.5	23.7	10.4	0.1
Gastritis	0.7	0.0	0.0	0.7	13.8	18.5	13.8	0.4
Hematological disorder	1.4	0.0	0.0	0.6	0.8	1.3	1.1	0.0
Hemophagocytic syndrome	0.0	0.0	0.0	0.6	5.2	7.3	5.3	0.4
Hepatitis	1.0	0.2	0.0	0.3	4.2	5.6	2.6	0.1
Hyperthyroidism	0.8	0.8	0.0	0.5	17.4	29.1	26.2	0.0
Hypopituitarism	4.0	0.6	0.0	2.4	57.6	67.4	16.6	0.5
Myositis/Rhabdomyolysis	1.1	0.2	0.0	0.4	3.1	5.3	3.1	0.1
Hypothyroidism	1.1	1.2	0.1	0.5	18.2	29.4	34.9	0.1
Myasthenia gravis	4.8	1.0	0.1	2.7	19.4	37.9	20.2	0.1
Myocarditis	2.8	0.4	0.0	1.4	10.5	17.6	11.4	0.2
Nephritis/renal dysfunction	0.9	0.1	0.0	0.1	2.0	3.3	2.7	0.0
Neurological disorder	3.1	0.3	0.0	0.5	3.6	4.5	4.3	0.0
Pancreatitis	1.2	0.1	0.0	0.4	3.9	6.0	4.7	0.2
Pneumonitis	1.6	0.1	0.0	3.0	3.4	7.9	5.6	0.0
Rash	0.6	0.0	0.0	0.2	2.8	3.8	1.8	0.1
Thyroid dysfunction	2.9	1.1	0.0	1.6	28.7	35.9	21.1	0.0
Type 1 diabetes mellitus	4.3	0.5	0.0	0.8	13.1	29.9	15.0	0.1

The SOM package of R (version 4.1.2; R Foundation for Statistical Computing, Vienna, Austria) was used to run the SOM. Meanwhile, decision tree analysis was performed using the rpart package, a machine learning library in R, to create classification and regression trees.

Ethics approval

Ethics approval was not sought for this study because it was observational and involved no research subjects. All results of this study are based on publicly available data on the website of the Pharmaceuticals and Medical Devices Agency (PMDA) [24]. In addition, data in the JADER database were completely anonymized by the regulatory authorities in advance and made safely accessible.

## Results

**Figure 1:**
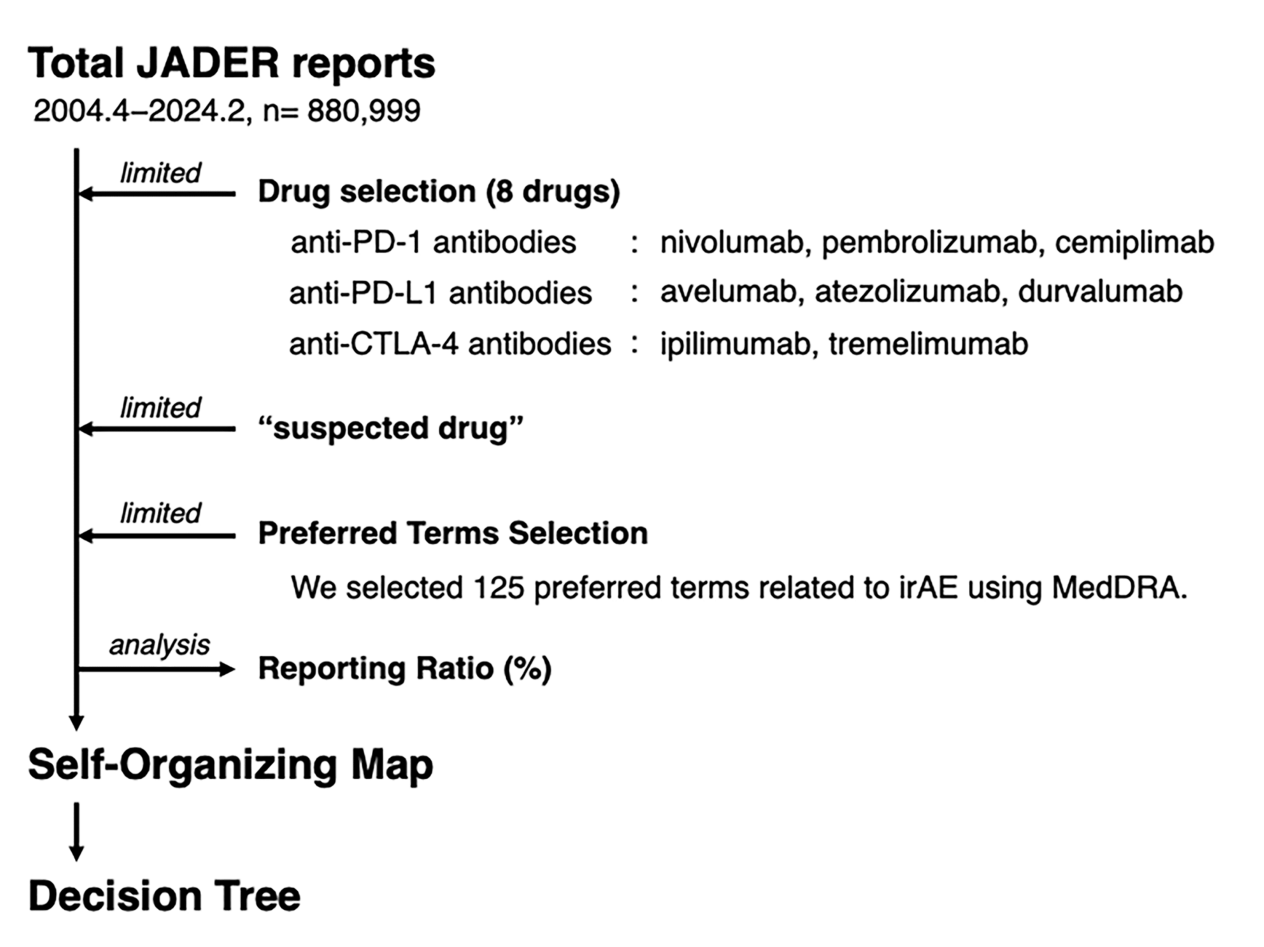
Flowchart depicting the process of data analysis JADER: Japanese Adverse Drug Event Report

The JADER database registered 880,999 reports published between April 2004 and February 2024 (Figure 1).

**Figure 2:**
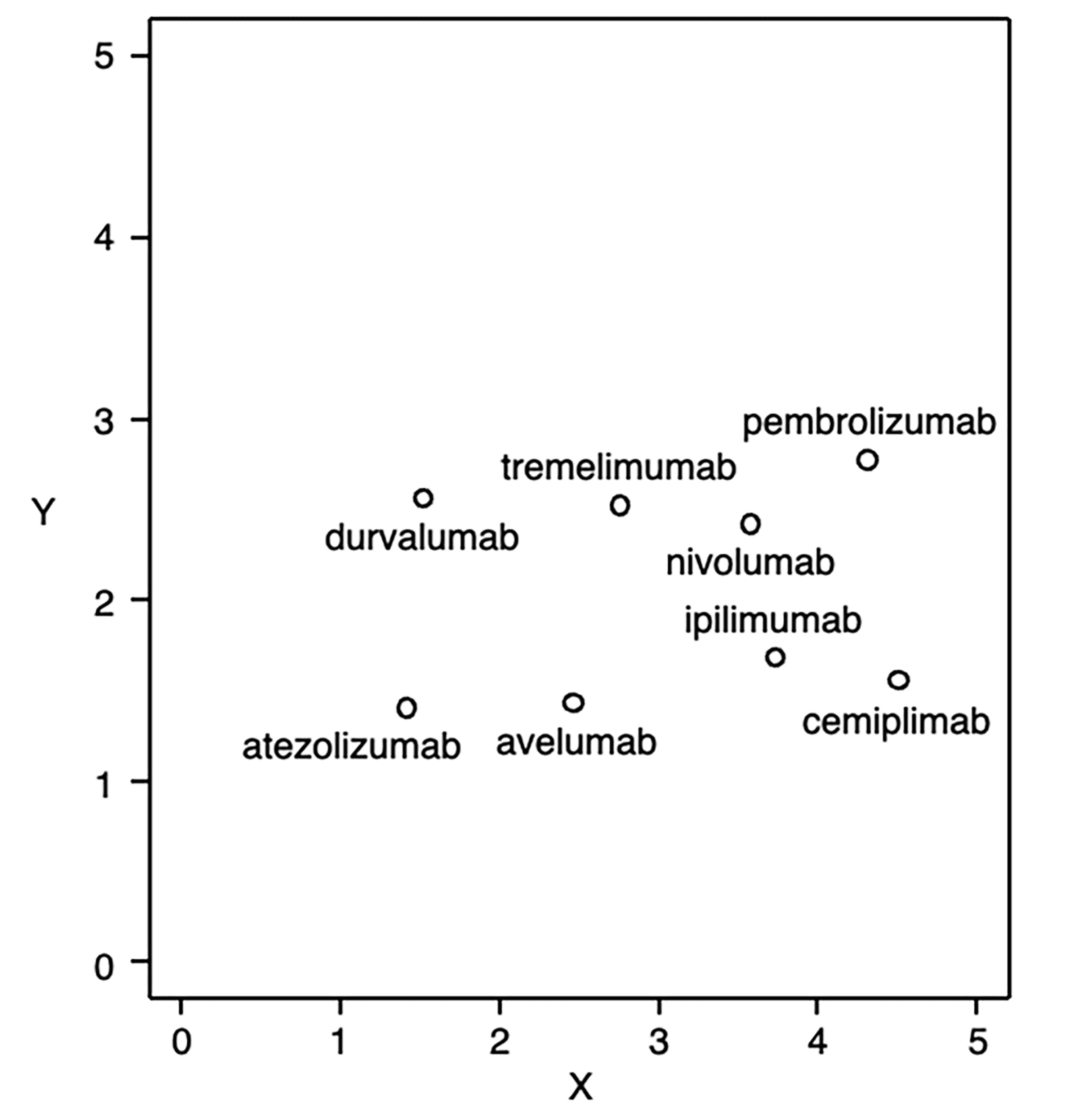
Self-organizing map of eight immune checkpoint inhibitors according to immune-related adverse events

The numbers of irAEs reported for atezolizumab, avelumab, cemiplimab, durvalumab, ipilimumab, nivolumab, pembrolizumab, and tremelimumab were 3797, 361, 17, 2554, 9315, 16,574, 11,487, and 196, respectively (Table 2). Demographic data are summarized in the Appendices. The irAE category reporting ratios (RRs) for each ICI are summarized in Table 3. The SOM results are shown as a 5 × 5 positioning map in Figure 2.

**Figure 3:**
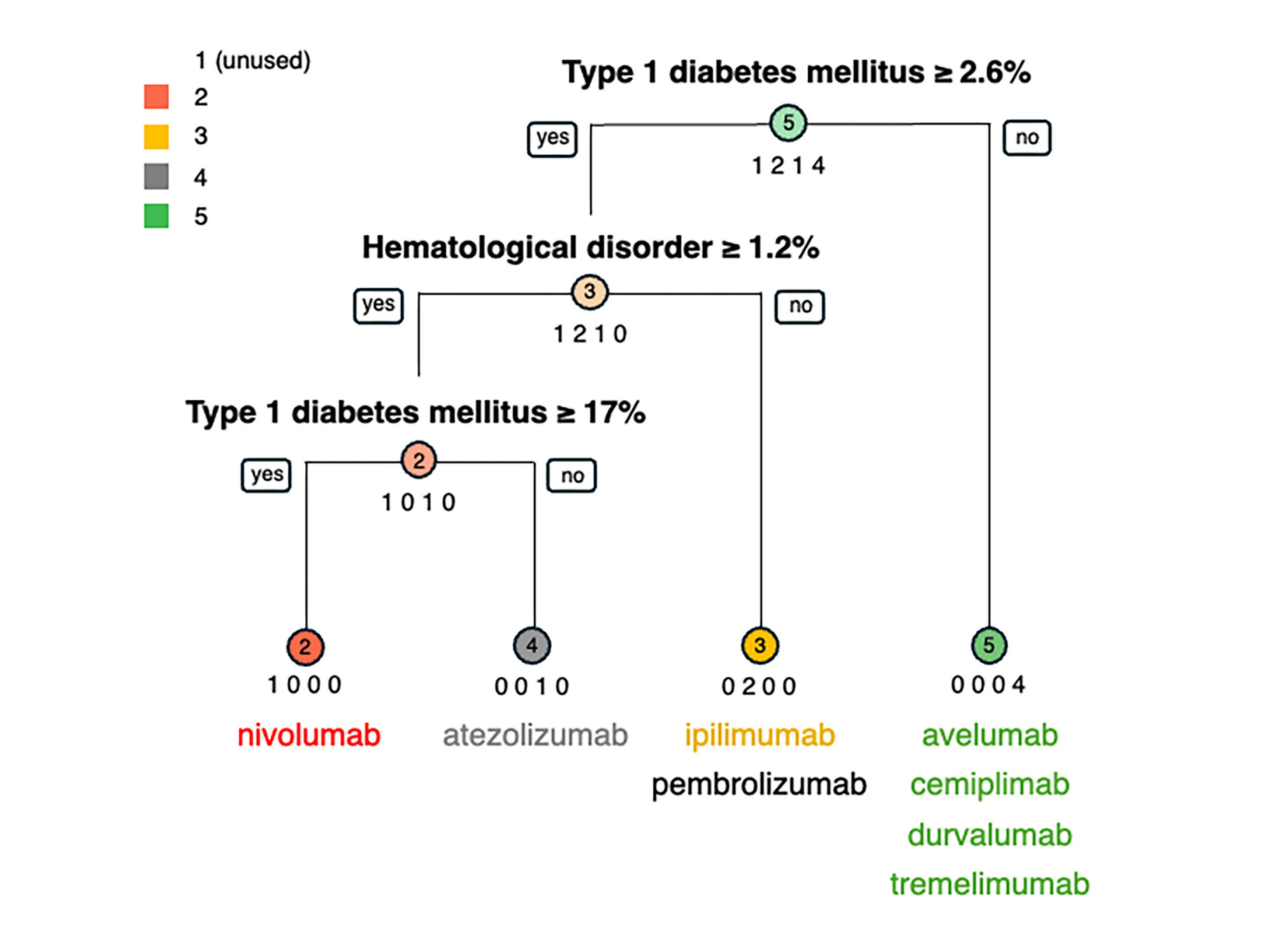
Decision tree of eight immune checkpoint inhibitors according to immune-related adverse events

The observation map of the SOM showed that adjacent units tended to be more similar to each other than to more distant units. However, it was not easy to interpret each unit clinically. Therefore, the number of units in layer 1 was kept at 24, whereas the number of units in layer 2 was set to 6, and the maps were arranged for competitive learning in a 6 × 1 format. In the decision tree analysis, we used the unit numbers assigned in the SOM as criterion variables and grew the 21 irAE categories as predictors (Table 3 and Figure 3).

The eight ICIs were divided into Unit 5 (avelumab, cemiplimab, durvalumab, and tremelimumab) for type 1 diabetes with an RR less than 2.6% and other ICIs for type 1 diabetes with an RR greater than 2.6%. Four ICIs with a type 1 diabetes mellitus RR of 2.6% or greater were further divided based on the RR for hematological disorders, with drugs with an RR for hematological disorders of less than 1.2% in Unit 3 (ipilimumab and pembrolizumab). ICIs with a hematological disorder RR of 1.2% or greater were divided based on the RR for type 1 diabetes mellitus, with drugs having an RR for type 1 diabetes mellitus of <17% in Unit 4 (atezolizumab) and ≥17% in Unit 2 (nivolumab).

## Discussion

In this study, we used SOM to visualize irAE profiles in ICIs. Furthermore, by combining decision tree analysis, we classified the eight ICIs from a perspective different from the immunological mechanism.

Multivariate analyses, such as principal component, factor, and discriminant analyses, have been conventionally used to compress multidimensional information and visualize it in low-dimensional maps. However, these methods, e.g., two-dimensional principal component analysis, have the disadvantage that only two major features can be considered, and information that cannot be represented is discarded. In contrast, with SOM, the differences between observations can be represented in as much detail as possible by increasing the number of units and expanding the map. In other words, SOM allows mapping with minimal loss of information. In the SOM shown in Figure 2, neighboring units show more similar features than distant units. Therefore, drugs with different irAE profiles were placed far from each other on the SOM.

In the SOM shown in Figure 2, each ICI resulted in a rough placement division based on the immune mechanism (Figure 2). The anti-PD-1 antibodies, nivolumab and pembrolizumab, have similar irAE profiles [26,27], and these two drugs were adjacent in the SOM in this study.

Cemiplimab, an anti-PD-1 antibody, and ipilimumab, an anti-CTLA-4 antibody, were adjacent in the SOM. Ipilimumab is indicated for the treatment of malignant melanoma, renal cell carcinoma, colorectal cancer, non-small cell lung cancer, malignant pleural mesothelioma, and esophageal cancer [28]. Cemiplimab is indicated only for cervical cancer, and ipilimumab and cemiplimab are administered to different patient groups [29].

In the SOM, nivolumab and ipilimumab, as well as durvalumab and tremelimumab, were adjacent to each other. Nivolumab and ipilimumab are used in combination in renal cell carcinoma, colorectal cancer, malignant pleural mesothelioma, and esophageal cancer [28,30]. Durvalumab and tremelimumab are administered in combination in non-small cell lung cancer and hepatocellular carcinoma [31,32]. Therefore, nivolumab and ipilimumab, as well as durvalumab and tremelimumab, are expected to have similar irAE profiles, respectively. Many studies have reported that the use of a combination of nivolumab and ipilimumab is associated with a high risk of irAEs [33-35]. Therefore, healthcare professionals should pay close attention to the risk of irAEs in patients receiving multiple ICIs in combination.

The decision tree analysis branched into four categories, from Units 2 to 5 (Figure 3). Among anti-PD-1, anti-PD-L1, and anti-CTLA-4 antibodies, drugs with the same immune mechanisms were classified across multiple units rather than within the same unit. ICIs can be classified according to the reported rates of hematological disorders and type 1 diabetes mellitus among irAEs. Type 1 diabetes mellitus is rare, occurring in less than 1% of cases [33]. The rate of type 1 diabetes mellitus was higher with anti-PD-1 antibody therapy than with anti-CTLA-4 antibody therapy [33,36]. In this study, the RR of type 1 diabetes was also high for anti-PD-1 antibodies other than cemiplimab, which has been on the market for only a short time and has few reported AEs. Different mechanisms and targets of anti-PD-1 and anti-CTLA-4 antibodies may partly explain the differences in the incidence of type 1 diabetes mellitus. This finding suggests that the clinical manifestations of irAEs can be divided into type 1 diabetes mellitus and hematological disorders, which may help healthcare professionals detect irAEs early.

Study limitations

The limitations of the current analysis should be acknowledged. We used data from the SRS JADER database. SRSs are passive reporting systems subject to confounding variables and numerous biases, such as under-reporting, over-reporting, and confounders due to comorbidities. Given these inherent problems, SRSs should not be used for true risk assessments using the RR [37,38]. The JADER database does not include detailed information such as clinical background, cancer type, stage, and chemotherapy regimen. Therefore, future studies should take these factors into account. Dosage information is entered in the JADER database, and there have been reports evaluating the association between the dosage of diabetes drugs [39], herbal medicines [40], and AEs [39,40]. However, the dosage of anticancer drugs varies with the regimen, and its evaluation in JADER was difficult; it may be desirable to use a dataset with detailed patient background, such as electronic medical record records, to associate ICI dosage and AE occurrence.

We could not clarify the factors contributing to the similarity in the irAE profiles of ipilimumab and cemiplimab. Cemiplimab should be evaluated carefully because it is new in Japan, launched in March 2023, and irAE reports are yet to be accumulated. Careful attention should be paid to interpreting the results of the decision tree analysis for ICIs with fewer reports such as avelumab, cemiplimab, and tremelimumab. For these agents, the accumulation of future reports may be expected to increase the predictive accuracy.

The relevance of SOM results to clinical applications can only be determined using extensive knowledge. A meaningful number of units can be formed if a healthcare professional can interpret the decision tree results as valid. Therefore, the validity of SOM results must always be considered based on their intended use. Despite the limitations of the SRS dataset, we believe that the AE SOM is useful for providing a comprehensive two-dimensional visualization of the similarity of AEs for diverse and complex drugs, and the discriminant method combined with decision tree analysis in this study is suggested.

We have already identified the reporting rate of irAEs by ICI, the reporting odds ratio, and the timing of onset of AEs for each drug using the JADER database dataset from April 2004 to June 2018 [26]. In addition, the age and concomitant medications that affect irAE have been investigated by association analysis [26]. Although the present results are for the period from 2004 to 2023, the data set is nearly identical, making comparisons easy, and the present results may have complementary value when combined with previous studies. Meanwhile, the U.S. Food and Drug Administration (FDA) Adverse Event Reporting System (FAERS) is the largest SRS in the world, with approximately 20 times as many reports as those in JADER [41]; many ICI AE investigations are reported using FAERS [42,43]. The application of SOM to FAERS is very interesting and deserves further study.

The SRS, including clinician reports of potential drug-related AE concerns, is a useful tool for drug safety surveillance and an essential data source for a comprehensive understanding of irAEs because it is based on actual data from clinical practice. Although analysis using SRS data is useful for obtaining the complete picture of irAEs, it must be interpreted with an awareness of its inherent limitations. Meanwhile, obtaining a complete picture of irAEs in ICI clinical trials remains a major challenge. This study suggests that AEs of type 1 diabetes mellitus and hematological disorders are useful indicators for identifying irAEs in ICI and may be useful for early intervention. However, the usefulness of the hypotheses derived in this study for predicting and managing irAEs in clinical practice by combining SRS data with SOM and decision tree analysis should be verified by clinical trial data and prospective cohort studies.

## Conclusions

Comprehensive analysis of the SRS database, combined with SOM and decision tree analysis, allowed us to systematically classify ICI for the first time based on the complex profile of irAEs in ICI treatment. The results of this study suggest the possibility of developing early prediction models for irAEs and contributing to the realization of personalized medicine. This study indicates the importance of utilizing real clinical data, such as JADER, in post-marketing drug safety surveillance.

## Appendices

**Table 4:** The number and reporting ratio of adverse event reports stratified by sex, age, and body weight

		Total	Atezolizumab		Avelumab		Cemiplimab		Durvalumab	
			Case (n)	Reporting Ratio (%)	Case (n)	Reporting Ratio (%)	Case (n)	Reporting Ratio (%)	Case (n)	Reporting Ratio (%)
Total		880999	6692	0.76	597	0.07	31	0.00	3568	0.40
Sex										
	Male	426126	4169	0.98	409	0.10	0	0.00	2372	0.56
	Female	412346	1606	0.39	167	0.04	31	0.01	613	0.15
Age (y.o.)										
	≤ 19 years	56286	2	0.00	0	0.00	0	0.00	2	0.00
	20–29 years	28199	10	0.04	0	0.00	0	0.00	1	0.00
	30–39 years	44134	39	0.09	1	0.00	1	0.00	11	0.02
	40–49 years	63248	188	0.30	19	0.03	3	0.00	79	0.12
	50–59 years	99583	572	0.57	38	0.04	8	0.01	271	0.27
	60–69 years	172094	1647	0.96	141	0.08	7	0.00	873	0.51
	70–79 years	207245	2538	1.22	250	0.12	1	0.00	1331	0.64
	80–89 years	107132	728	0.68	115	0.11	0	0.00	231	0.22
	90–99 years	16000	23	0.14	1	0.01	0	0.00	7	0.04
	≥ 100 years	356	3	0.84	0	0.00	0	0.00	0	0.00
Body weight (kg)										
	≤ 19	13295	0	0.00	0	0.00	0	0.00	0	0.00
	20−29	4038	1	0.02	0	0.00	0	0.00	0	0.00
	30−39	21356	92	0.43	6	0.03	2	0.01	33	0.15
	40−49	75999	444	0.58	49	0.06	0	0.00	195	0.26
	50−59	102024	709	0.69	71	0.07	1	0.00	424	0.42
	60−69	70719	632	0.89	80	0.11	0	0.00	368	0.52
	70−79	30087	262	0.87	40	0.13	0	0.00	141	0.47
	80−89	9823	75	0.76	8	0.08	0	0.00	45	0.46
	90−99	3248	21	0.65	7	0.22	0	0.00	10	0.31
	100−109	1162	5	0.43	2	0.17	0	0.00	2	0.17
	110−119	436	1	0.23	2	0.46	0	0.00	0	0.00
	≥ 120	800	2	0.25	1	0.13	0	0.00	3	0.38

**Table 5:** The number and reporting ratio of adverse event reports stratified by sex, age, and body weight (continued)

		Ipilimumab		Nivolumab		Pembrolizumab		Tremelimumab	
		Case (n)	Reporting Ratio (%)	Case (n)	Reporting Ratio (%)	Case (n)	Reporting Ratio (%)	Case (n)	Reporting Ratio (%)
Total		12016	1.36	22944	2.60	17380	1.97	298	0.03
Sex									
	Male	8604	2.02	16691	3.92	10142	2.38	232	0.05
	Female	3054	0.74	5698	1.38	6637	1.61	52	0.01
Age (y.o.)									
	≤ 19 years	13	0.02	29	0.05	5	0.01	1	0.00
	20–29 years	38	0.13	77	0.27	20	0.07	0	0.00
	30–39 years	174	0.39	277	0.63	192	0.44	4	0.01
	40–49 years	707	1.12	1121	1.77	686	1.08	8	0.01
	50–59 years	1761	1.77	3014	3.03	2014	2.02	33	0.03
	60–69 years	3423	1.99	6919	4.02	4617	2.68	53	0.03
	70–79 years	4533	2.19	8584	4.14	6369	3.07	124	0.06
	80–89 years	954	0.89	1944	1.81	1736	1.62	46	0.04
	90–99 years	13	0.08	38	0.24	70	0.44	4	0.03
	≥ 100 years	0	0.00	0	0.00	0	0.00	0	0.00
Body weight (kg)									
	≤ 19	4	0.03	4	0.03	1	0.01	0	0.00
	20−29	4	0.10	6	0.15	5	0.12	0	0.00
	30−39	128	0.60	363	1.70	230	1.08	6	0.03
	40−49	774	1.02	1918	2.52	1107	1.46	18	0.02
	50−59	1339	1.31	3120	3.06	1864	1.83	52	0.05
	60−69	1182	1.67	2396	3.39	1336	1.89	36	0.05
	70−79	570	1.89	1051	3.49	532	1.77	15	0.05
	80−89	170	1.73	301	3.06	128	1.30	7	0.07
	90−99	57	1.75	86	2.65	39	1.20	1	0.03
	100−109	9	0.77	22	1.89	15	1.29	1	0.09
	110−119	10	2.29	15	3.44	5	1.15	0	0.00
	≥ 120	5	0.63	7	0.88	27	3.38	0	0.00
